# Disturbance Regimes Drive The Diversity of Regional Floristic Pools Across Guianan Rainforest Landscapes

**DOI:** 10.1038/s41598-018-22209-9

**Published:** 2018-03-01

**Authors:** Stéphane Guitet, Daniel Sabatier, Olivier Brunaux, Pierre Couteron, Thomas Denis, Vincent Freycon, Sophie Gonzalez, Bruno Hérault, Gaëlle Jaouen, Jean-François Molino, Raphaël Pélissier, Cécile Richard-Hansen, Grégoire Vincent

**Affiliations:** 1 AMAP, IRD, Cirad, CNRS, INRA, Université de Montpellier, Montpellier, France; 2 ONF Guyane, Département Recherche et Développement, Réserve de Montabo, 97307 Cayenne, French Guiana; 3 ONCFS, Direction de la Recherche et de l’Expertise, Campus agronomique, 97379 Kourou, French Guiana; 4 CIRAD, Forêts et Sociétés, Campus de Baillarguet, 34398 Montpellier, France; 5 IRD, Amap, Herbier de Cayenne, 97307 Cayenne, French Guiana; 6 CIRAD, EcoFoG, 97379 Kourou, French Guiana; 7 AgroParisTech, EcoFoG, 97379 Kourou, French Guiana

## Abstract

Disturbances control rainforest dynamics, and, according to the intermediate disturbance hypothesis (IDH), disturbance regime is a key driver of local diversity. Variations in disturbance regimes and their consequences on regional diversity at broad spatiotemporal scales are still poorly understood. Using multidisciplinary large-scale inventories and LiDAR acquisitions, we developed a robust indicator of disturbance regimes based on the frequency of a few early successional and widely distributed pioneer species. We demonstrate at the landscape scale that tree-species diversity and disturbance regimes vary with climate and relief. Significant relationships between the disturbance indicator, tree-species diversity and soil phosphorus content agree with the hypothesis that rainforest diversity is controlled both by disturbance regimes and long-term ecosystem stability. These effects explain the broad-scale patterns of floristic diversity observed between landscapes. In fact, species-rich forests in highlands, which have benefited from long-term stability combined with a moderate and regular regime of local disturbances, contrast with less diversified forests on recently shaped lowlands, which have undergone more recent changes and irregular dynamics. These results suggest that taking the current disturbance regime into account and including geomorphological stratifications in climate-vegetation models may be an effective way to improve the prediction of changes in species diversity under climate change.

## Introduction

The Intermediate Disturbance Hypothesis (IDH) that predicts a humped diversity-disturbance relationship is at the same time inspiring for conservation policies^1,2^ and subject to scientific controversy^3,4^. The theory suggests that in highly diverse ecosystems, like the tropical rainforest, where competitive exclusion prevails in late successional processes^5^, an intermediate disturbance regime, i.e. of moderate intensity and/or frequency, locally reduces inter-individual competition for resources and thus allows less competitive species to avoid exclusion and to maintain in the community^6^. There are empirical supports for IDH to locally maintain tropical rainforest in a non-equilibrium dynamics enhancing species diversity through gap-phase regeneration processes and secondary successions (e.g. refs^7–10^). However, at larger scales corresponding to the extent of forest management options the diversity-disturbance relationship is actually not so clear.

Spatio-temporal variations in resource availability, niche diversity or immigration fluxes (related e.g. to mass effects) may blur the expected diversity patterns that are observed at local scale (e.g. refs^11,12^). For instance, Stropp and colleagues^13^ inferred from wood density data across a network of 1-ha forest plots in Amazonia, that the frequency of disturbances was the main process driving local diversity, but not regional diversity, which they found more correlated to proxies of paleoclimatic stability and long-term ecosystem dynamics. These findings suggest that both local- and large-scale spatio-temporal dynamic processes interact in shaping the current pattern of rainforest tree species diversity, and should be accounted for when anticipating future changes^14,15^. A related question is to develop efficient indicators capable of faithfully capturing rainforest large-scale disturbance regimes^16^.

In this paper we used an extensive multi-disciplinary forest inventory conducted between 2006 and 2015 across French Guiana (South America) in order to test whether regional patterns of tree and understory diversity depend on large-scale disturbance regimes. We used one-hundred-and-eleven 3 km-long transects in 33 different sites spread over all French Guiana territory to develop robust indicators of regional species diversity. We computed diversity indices for trees and understory vegetation at each site (average of 20 ha inventoried per site representing tens of km²) using rapid assessment methods. The disturbance intensity was measured using the relative frequency of Pourouma and Cecropia (Urticaceae) species, which are strict early-successional pioneers whose abundance was demonstrated to depend on canopy gap frequency as assessed from aerial LiDAR data at two test sites. We also measured soil properties on 450 soil profiles to directly assess nutrient availability, niche diversity and long-term ecosystem stability. Geospatial data were also used to account for environmental drivers of regional diversity such as rainfall^13^ or geomorphology^17^, which have already shown to be explanatory of broad scale floristic patterns in French Guiana^18,19^.

## Results

### Tree diversity is affected by geomorphology and rainfall

**Figure 1:**
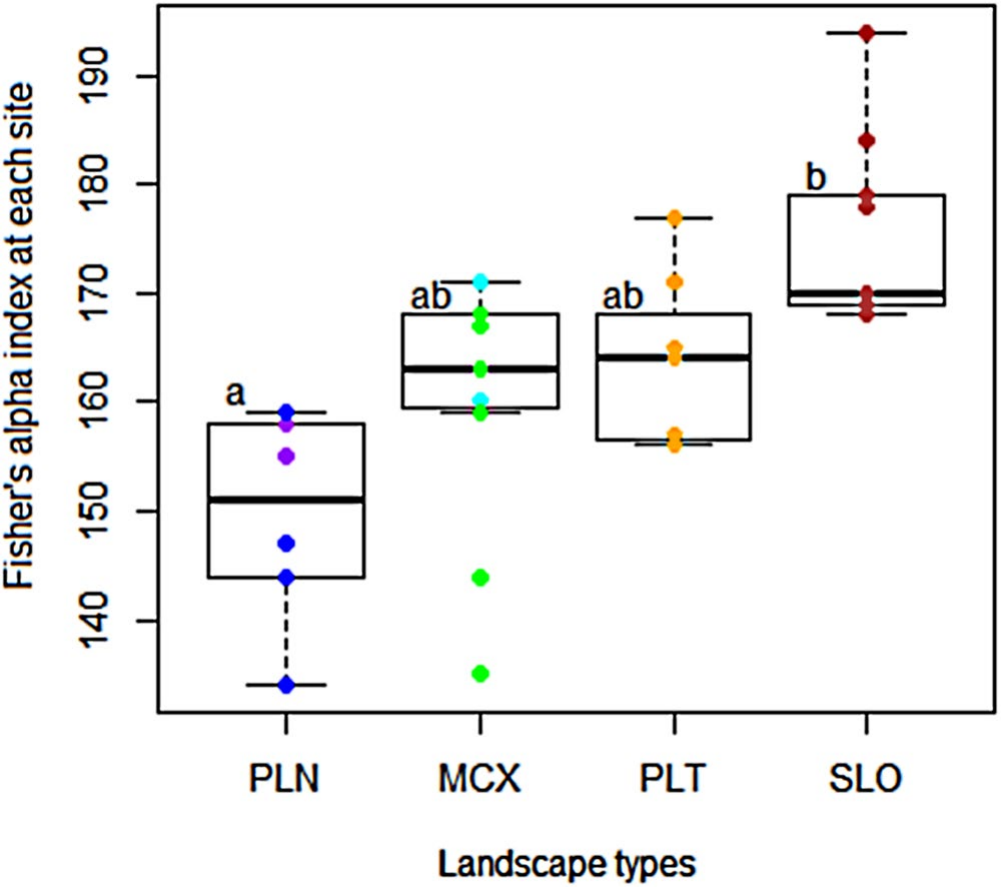
Variation in tree species diversity (site scale) according to geomorphological landscape categories. PLN stands for coastal plains (in blue) and inland plains (in purple), MCX stands for multiconvex reliefs with hills (in green) and large valleys (in cyan), PLT stands for tablelands (in orange) and SLO stands for all-slope relief (in brown). Letters indicate between-group significant differences based on the HSD test.

Estimated Fisher’s alpha diversity index in the study regions ranged from 134 to 194 among sites and depended significantly on the type of relief (F-statistic = 8.771, DF = 3, adj-R² = 0.42, P = 0.00027, Fig. 1). The highest values of Fisher’s alpha were observed on small mountains (SLO < 800 m above sea level), and the lowest values were observed on coastal and inland plains (PLN); intermediate levels of diversity were observed in other types of landscape (i.e., tablelands or hilly reliefs). Using variogram analysis, we found no significant autocorrelation that could explain part of the regional variation in tree species diversity (Supplementary Fig. S1). Elevation and rainfall were the main environmental factors explaining this pattern (F-statistic = 13.66, DF = 3, adj-R² = 0.54, P < 10^−5^), with a linear positive effect of elevation (t-value = 4.866, P < 0.0001) and a quadratic effect of rainfall (t-value = 2.055, P = 0.049 for the first order, t-value = −1.925, P = 0.0641 for the second order). Their combination provided the best explanatory model for regional diversity.

### Frequency of short-lived pioneers is a better proxy of disturbance than the frequency of all pioneer species

**Figure 2:**
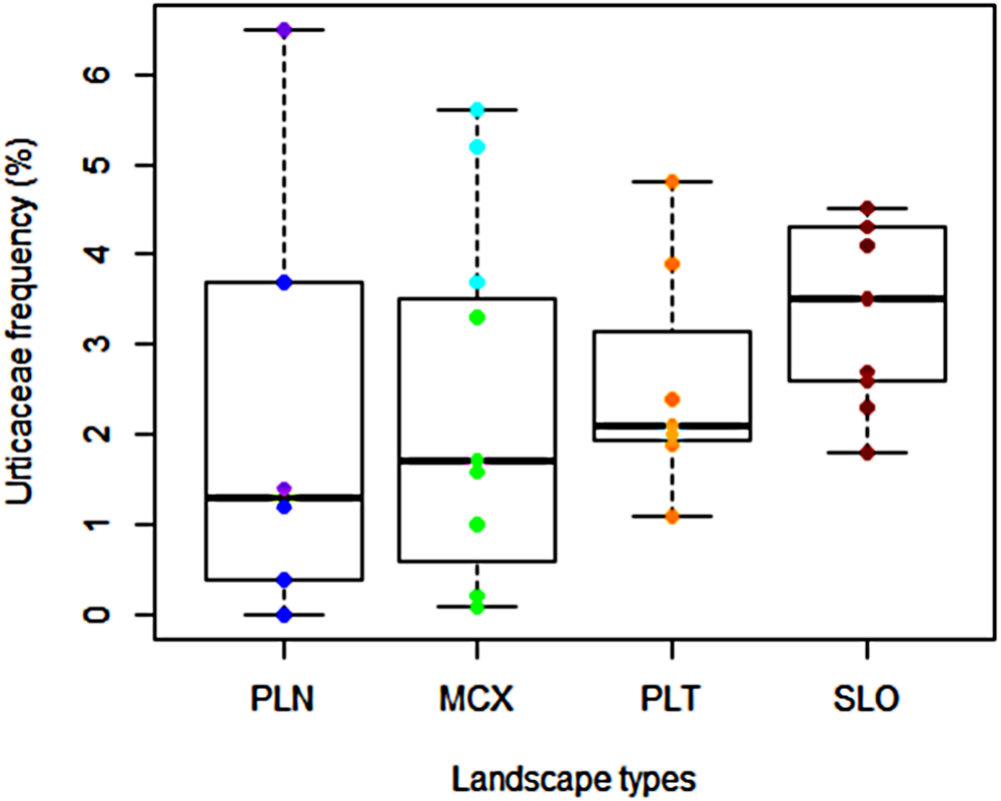
Variation in Urticaceae frequency expressed as the proportion of stems (site scale) according to geomorphological landscape categories. The different colours indicate the categories of relief (see legend of Fig. 1).

**Figure 3:**
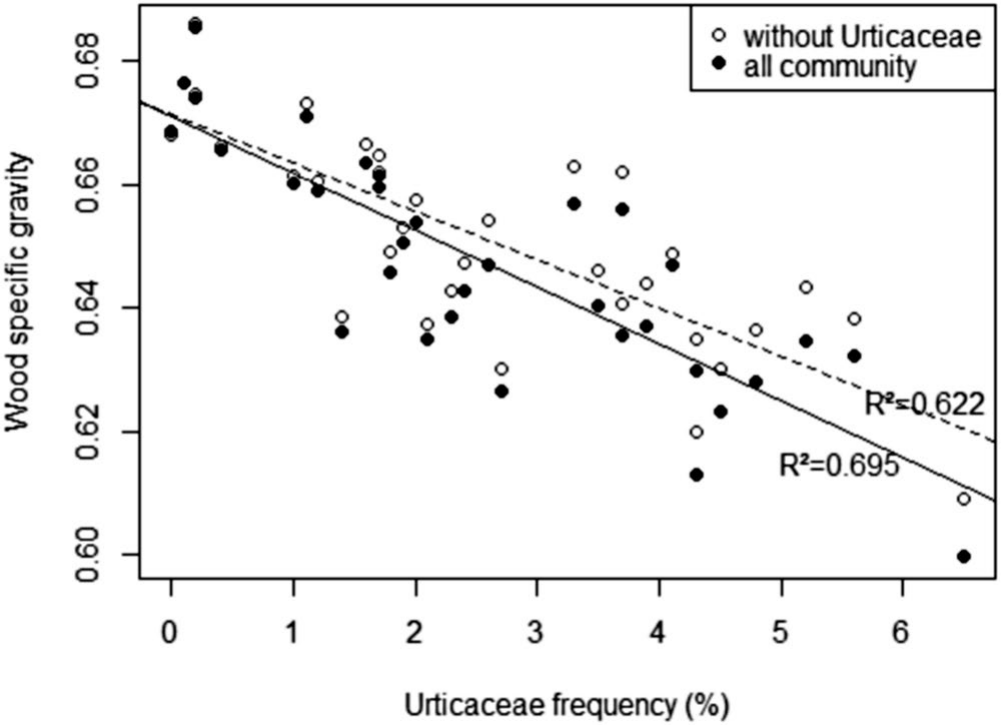
Correlation between mean wood specific gravity and Urticaceae frequency at the site scale. The black circles indicate the WSG of the whole-tree community, and the empty circles indicate mean WSG after excluding Urticaceae.

The frequency of all pioneers in large tree communities, which is usually used as a disturbance indicator, varied from 1% to 12.6% among sites, whereas the frequency of short-lived and early successional pioneers belonging to the Urticaceae family varied from 0% to 6.5% (Fig. 2). Mean Urticaceae frequency increased from plains (PLN = 1.2%) to small mountains (SLO = 3.5%), whereas its variance decreased along the same gradient. The community mean of wood specific gravity (WSG), which is a classical indicator of a disturbance regime, was negatively correlated with the frequency of Urticaceae (Fig. 3, r = −0.833, t = −8.3974, DF = 31, P < 10^−8^) but less strongly correlated with the frequency of all pioneers (r = −0.469 – P < 0.01). We checked that the relationship between WSG and the frequency of Urticaceae was still valid after excluding Urticaceae trees from the WSG computation (Fig. 3, r = −0.789, t = −7.1471, DF = 31, P < 10^−7^). At the two sites at which LiDAR data enabled quantification of canopy gaps (based on top of canopy height – see the Materials and Methods section), we found a significant correlation between Urticaceae frequency and the gap fraction computed for 500-m buffer zones around the line transects (DF = 3, r = 0.986 for gaps defined by canopy height < 2 m, r = 0.994 with canopy height < 5 m and r = 0.969 with canopy height < 11 m - p < 0.005 in all cases). By contrast, we found no significant correlation with the frequency of all the pioneer species (r = 0.543 to 0.761 and p > 0.2 for all heights – see Supplementary Fig. S2).

### Disturbance regime and long-term stability significantly explain broad-scale diversity patterns

Applying Bayesian averaging models to the entire dataset (including sites for which no soil analyses were available) or to a subsample of 21 sites for which complete soil analyses were available, we found that disturbance (DI approximated by Urticaceae frequency) was a more important factor than habitat diversity (HD approximated by soil diversity, mean slope, elevation range), resource availability (RA approximated by soil properties), or long-term climatic stability (LS approximated by rainfall, elevation and geographical coordinates) in explaining tree diversity.

**Figure 4:**
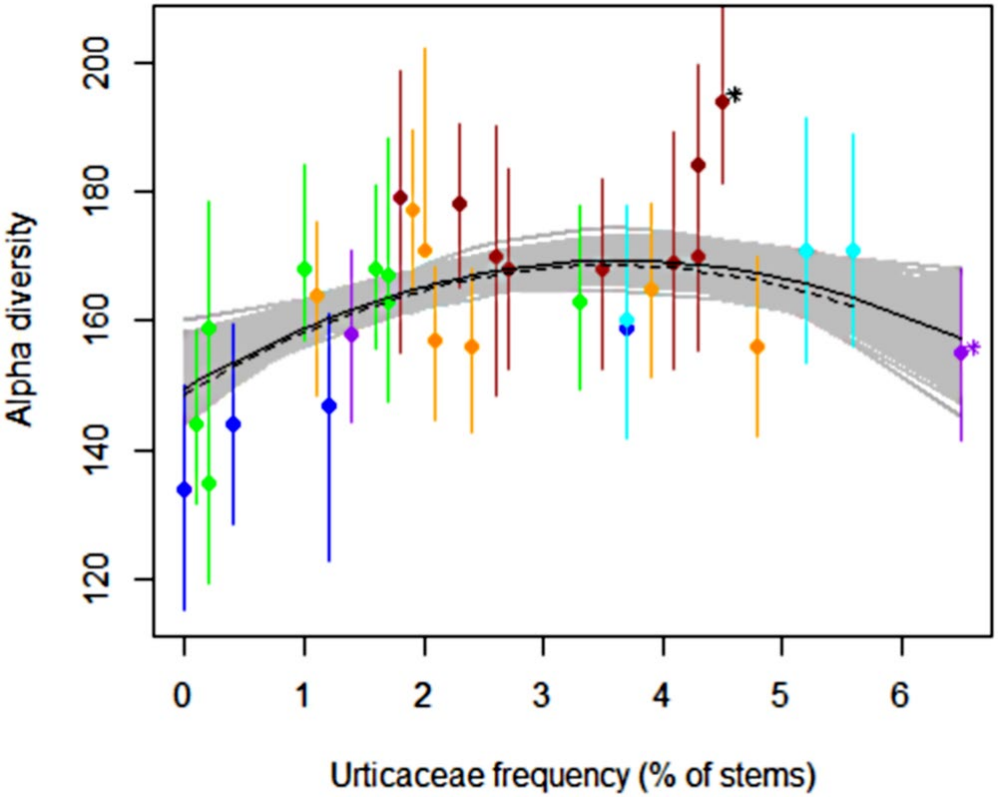
Relationship between Urticaceae frequency (proportion of stems) and Fisher’s alpha diversity index for the whole dataset (33 sites). Full circles represent mean Fisher’s alpha values, and vertical lines show the range of Fisher’s alpha simulations. Colours show the relief categories (see legend of Fig. 1). The solid black line shows the disturbance effect on Fisher’s alpha at maximum likelihood. The grey portion indicates the confidence intervals at 95% from the Bayesian inference. The dashed lines indicate the same effect and confidence intervals if two extreme sites (indicated by *) were removed.

Indeed, in the first run including the entire dataset, Urticaceae frequency and its quadratic effect best explained Fisher’s alpha diversity (posterior inclusion probabilities, i.e., PIP, 0.876 for Urticaceae and 0.803 for the quadratic term - see Supplementary Fig. S3). Elevation and rainfall, two variables assumed to approximate LS, had an intermediate PIP (0.606 and 0.383, respectively), whereas the other factors had a low PIP (0.353 to 0.07). The linear model resulting from the stepwise selection included the four variables with highest PIP and accounted for 72% of the global variance (F-statistic = 18.05, DF = 28, adj-R² = 0.6806, P < 10^−6^ – see Table 1). The DI effect captured by Urticaceae frequency (entered as two factors via a linear and a quadratic component) accounted for 43% of the total variance with a clear maximum for intermediate values (Fig. 4). Removing the extreme sites (the most diversified and the most disturbed – indicated by stars in Fig. 4) did not alter either the shape or the strength of the relationship between diversity and Urticaceae (F-statistic = 12.42, DF = 26, adj-R² = 0.6036, P < 10^−5^). However, it should be noted that the hump-shaped relationship was mainly driven by the first part of the curve, with a marked increase in diversity from 0% to 3% of Urticaceae frequencies, whereas the decline in the second part of the curve was less pronounced. Replacing the quadratic term by a free power parameter in a Bayesian inference approach led to a power factor ranging from 1.1 to 2.4 (median = 1.28), which was associated with a negative and co-varying coefficient that mitigated the hump of the curve but confirmed the unimodal pattern, especially the rising, initial part of the curve (Fig. 4).

**Table 1:** Factors selected in the best models for diversity prediction: estimate of the parameter and t-test for significance and f-value from anova (***P < 0.001, **P < 0.01, *P < 0.05).

Model	Variables	Estimate	T value	F value
Best model with 33 sites without soil properties	Rainfall	0.017733	3.187**	14.121 ***
	Elevation	0.054622	4.644***	21.563 ***
	Frequency of Urticaceae	10.839859	4.077***	17.027 ***
	Frequency of Urticaceae^²^	−1.480906	−3.452**	19.472 ***
Best model with 21 sites including soil properties	Frequency of Urticaceae	11.7057	18.270*	3.6012 ns
	Frequency of Urticaceae^²^	−1.6270	−2.150*	4.6237*
	Phosphorus	−3.5211	−1.309	7.4309*

**Figure 5:**
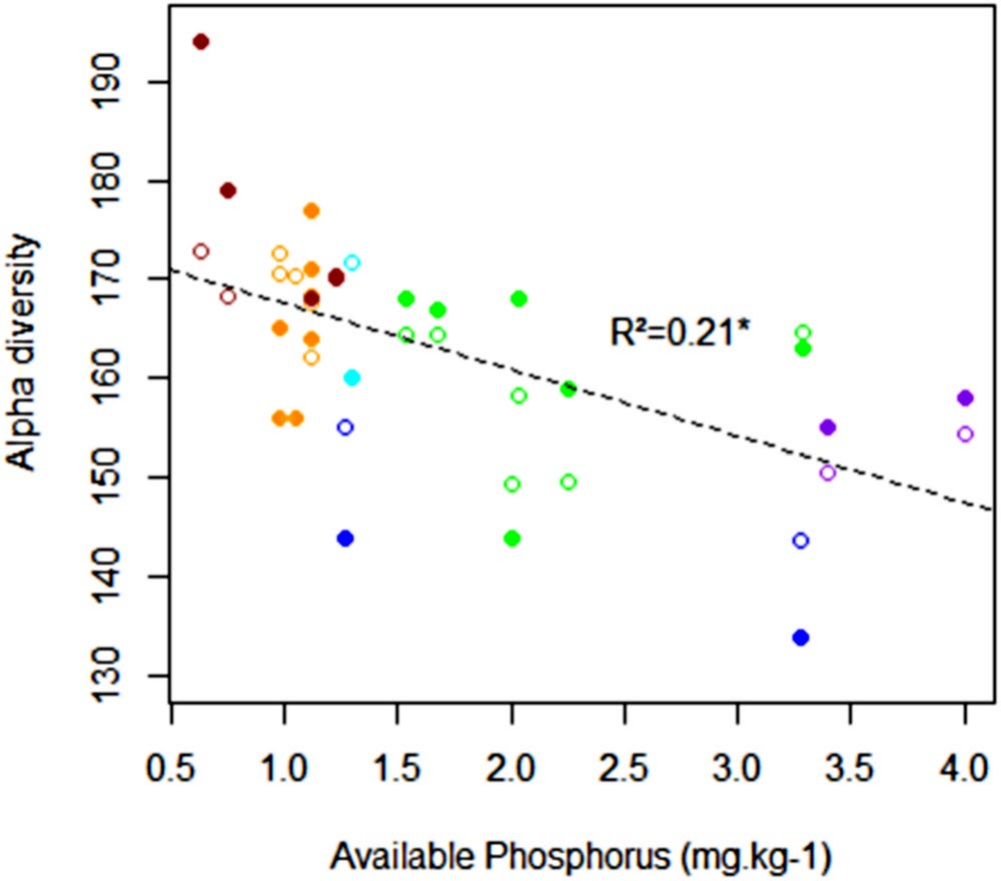
Correlation between alpha diversity at the site scale and Bray-2 extractable phosphorus. Full circles represent measurements, and empty circles represent the values predicted by the best predictive model, including phosphorus and disturbance effects (first order and second order for Urticaceae frequency). Colours indicate the relief categories (see legend of Fig. 1), and the dashed line represents the effects of phosphorus alone.

In the second run, when soil properties and rainfall were included as predictive factors to approximate resource availability instead of all other environmental proxies, Urticaceae frequency and its quadratic effect still had the highest PIP (Fig. S3 - PIP = 0.555 and 0.483, respectively), followed by Bray-2 extractable phosphorus (PIP = 0.362) and clay content (PIP = 0.335). However, the effect of clay content was not retained after stepwise selection in the linear model, and Bray-2 extractable phosphorus was shown to have a negative but non-significant effect on Fisher’s alpha diversity (Table 1 - Fig. 5).

### Tree and understory diversity share the same trends

Understory diversity and tree diversity were significantly and positively correlated, suggesting that understory vegetation shares the same drivers as those of tree communities (r = 0.86, t = 5.2798, DF = 10, P = 0.00036 - Supplementary Information Fig. S4). However, we found no significant relationship between understory diversity and the frequency of Urticaceae. By contrast, a slight positive correlation between understory diversity and the quadratic frequency of all pioneer species (r = 0.6971, t = 3.0746, DF = 10, P = 0.01175) and a negative correlation with Bray-2 extractable phosphorus (r = −0.7374, t = −2.6741, DF = 6, P = 0.03682) were observed.

## Discussion

**Figure 6:**
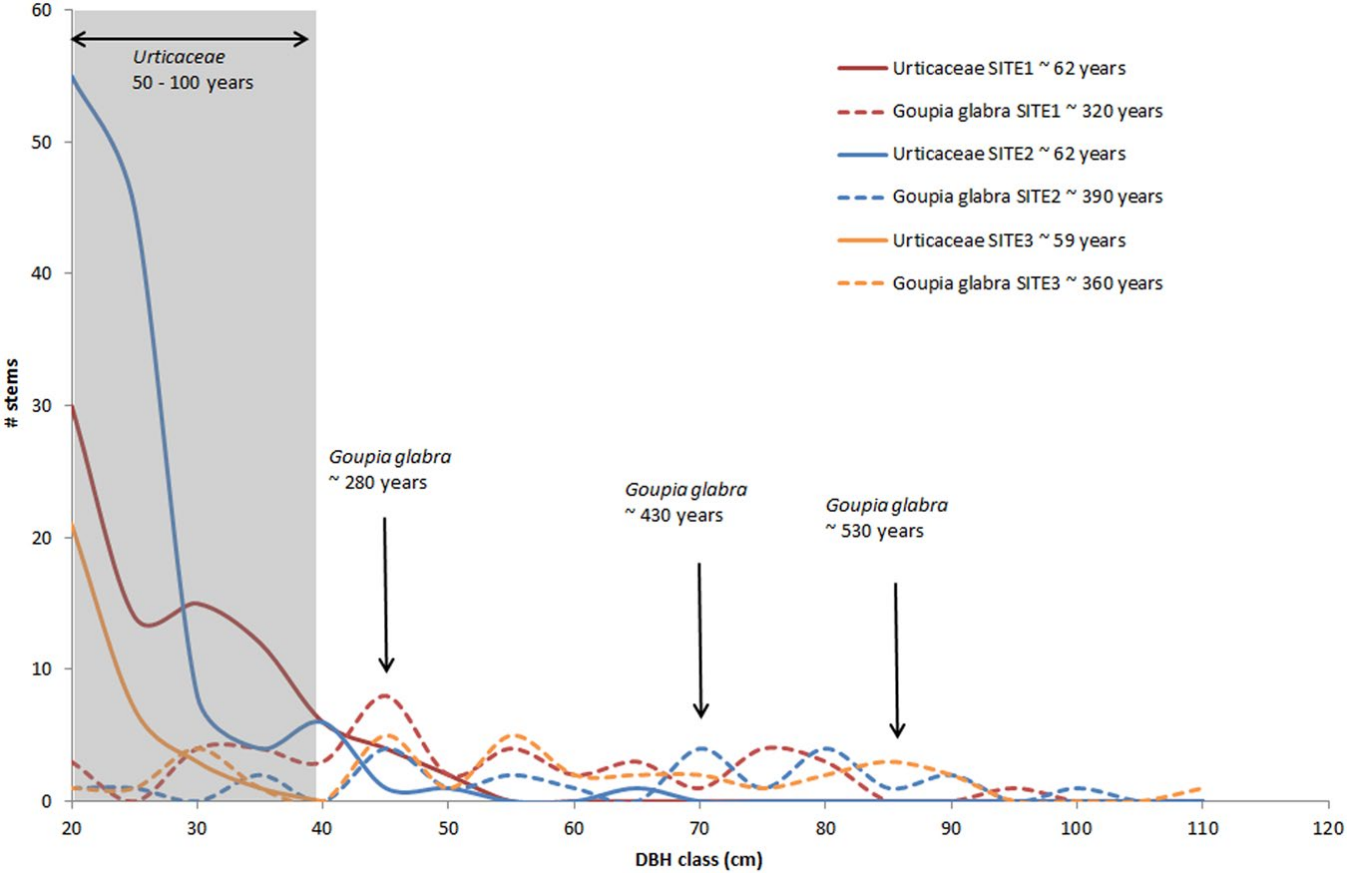
Examples of diameter distribution of short-lived pioneers (solid lines: Urticaceae) and a long-lived pioneer (dashed lines: *Goupia Glabra*) at 3 sites. Sites 1 and 2 have a similar pioneer frequency (3.6%) but different Urticaceae frequency (1.6% and 3.5%, respectively). Site 3 has a similar Urticaceae frequency as does site 2 (3.7% vs 3.5%) but a higher pioneer frequency (11.5%). The estimated mean ages of the different cohorts (indicated with arrows) were inferred from the growth rate and the methodology described previously^29^. The mean ages of the all populations were estimated from the diameter distributions and are listed in the key in the top-right corner of the figure.

In all Neotropical forest, within communities of large trees, the short-lived pioneer guild mostly comprises *Pourouma* spp. and *Cecropia* spp. (Urticaceae), which are widespread and very easy to recognise^20,21^. This group of species belonging to the Urticaceae family is a simple but very reliable indicator of a disturbance regime in rainforests. The frequency of these Urticaceae species is directly related to the canopy openness measured by LiDAR over large areas (~10 km²), including recent treefall gaps and frequent branch falls^22^ but also patches of low vegetation, including bamboo thickets, herbaceous swamps, bare outcrops and large areas disturbed by serious but rare disturbances such as landslides and blowdowns. Thus, in the absence of long-term series at the landscape scale, Urticaceae can be used as a simple but reliable marker for short-term disturbance regimes, i.e., half a century to a century depending on the lifetime of the species^21^. Interestingly, the frequency of Urticaceae was closely correlated with the mean wood density of the surrounding community (Fig. 3), a trait usually associated with long-term forest dynamics^23^. Hence, we can assume that the frequency of short-lived pioneer tree species such as *Cecropia* and *Pourouma* not only is a good proxy of recent disturbances, which enables these species to develop in a forest community, but also reflects the long-term disturbance regime of the forest community we studied (i.e., forest dynamics). By focusing on a few widely distributed, easily recognisable species, this new disturbance indicator is easier to measure at the landscape scale than mean wood density or the frequency of the whole pioneer guild. Moreover, focusing on a homogeneous pool of functionally similar widespread species makes the indicator more robust and less heteroscedastic than the usual whole pioneer guild indicator. Indeed, mixing species with different life-history strategies^24^ and growth rates^25^ such as short-lived species (e.g., *Cecropia* spp. and *Pourouma* spp.) with long-lived pioneer species (e.g., *Bagassa guianensis*, *Simarouba amara*, *Goupia glabra* or *Ficus* spp.) in the same indicator may lead to inconsistencies in the evaluation of disturbances (Fig. 6). Mixing short-lived and long-lived species may not be problematic when studies focus on saplings and recruitment in response to recent local disturbances^7^, but this mixing may be confusing in studies of uneven-aged tree communities that may have undergone irregular disturbance regimes (i.e., stages of intense disturbance alternating with stages of little disturbance, as was the case for *Goupia glabra* populations in the medium term in Fig. 6). Other short-lived, widely dispersed pioneer species, such as *Miconia* and *Vismia*, could also be added to the proposed indicator to form a complete group of “early successional short-lived” indicator species. *Vismia* species in particular are known as early successional pioneer species linked to fire disturbances in the Amazon forest^26^. *Vismia* species were not present in our records but could be added to *Pourouma* and *Cecropia* to build a more generic indicator of disturbance intensity for all Neotropical forests^27^.

The hump shape of the relationship between diversity and Urticaceae frequency agrees with the IDH effect, as local diversity at the landscape scale significantly increases when either sporadic, strong disturbances (e.g., windthrow or small blowdowns) or constant moderate disturbances occur, but decreases slightly when such disturbances become more frequent and/or stronger. This result extends the conclusions of Molino and Sabatier^7^ to the landscape scale, as these authors previously demonstrated a clear IDH effect at the local scale. We also checked the assumption that the pool diversity of regional species includes the long-term history of the disturbance regime^28^, as suggested by the relationship between Urticaceae frequency and wood specific density. Using a direct surrogate for disturbance, we demonstrated that the disturbance-diversity relationship is in agreement with the IDH and that disturbances are important drivers of diversity maintenance in natural ecosystem functioning^29^, particularly in mature and relatively stable rainforest ecosystems.

Previous studies conducted in wet evergreen African forests (Ghana) failed to detect this disturbance-driven effect^10^. Two hypotheses may explain this inconsistency. First, the disturbance indicator used by Bongers^10^, who observed the mixing of long-lived and short-lived pioneer species, may be not sufficiently reliable, as discussed above. Second, African wet forests and Neotropical rainforests have experienced distinct ecological histories that explain divergences in their present functioning^30^ and result in substantially richer local tree communities in Amazonia^31^. Paleo-climatic studies indeed suggest that the areal extent of African rainforests may have been more reduced during the Last Glacial Maximum and that the rainforest in Ghana is a younger ecosystem than is the Guianan rainforest^32^. Moreover, many hints, such as the isotopic profile of soil organic carbon, suggest recent forest colonisation or forest-savannah cycles in West and Central Africa^33,34^, whereas the Guianan forest has proven to be more resilient during the Holocene and not to have undergone significant regression^35^. Moreover, climates that prevail in most of the African lowlands are not equivalent and probably drier during all the quaternary, than in Neotropical forest, inducing “youngest” and poorest forest ecosystems in Africa than in Amazonia^31^. We can thus assume that IDH plays a significant role in explaining the regional diversity pattern in very old forest ecosystems where resource availability is reduced by soil weathering and where meta-communities may have accumulated a high level of species richness^36^, but that the IDH is a less important driver in younger ecosystems. In ancient ecosystems, the mechanisms underlying the IDH are clearly expressed and evident through relevant, broad-scale data. Conversely, these mechanisms may play a more limited role in driving regional patterns of diversity in more recent or unstable forest ecosystems, where regional species pools display less richness^31^ and are still limited by low migration processes and incomplete succession stages. In fact, in the youngest geomorphological landscapes (such as recent coastal plains in French Guiana or large alluvial floodplains in Africa) recently colonised by forests, young soils have high resource availability albeit under more constraining conditions for vegetation due to limited drainage. The resulting strong environmental filtering may drastically increase the niche effect^37^ and reduce inter-species competition, thereby downplaying the effect of IDH.

The negative effect of Bray-2 extractable phosphorus on diversity and the relationship between diversity patterns and the different categories of relief reinforce the interpretation that long-term ecosystem stability and disturbances are the main drivers of regional species pool diversity^13^. This negative relationship appears to be in opposition with the CSR theory^24^, which predicts that in less productive habitats (i.e., low resource availability) resource depletion limits the range of strategies at the disposal of plants, especially strategies linked to competitiveness, and consequently reduces the potential diversity. However, in our study, Bray-2 extractable phosphorus content did not exceed 4 mg.kg^−1^ in any of our soil profiles, which is very low. Soils are mainly Ferralsols, i.e., old weathered soils that require a very humid permanent tropical climate to develop^38,39^. In this context, the Bray-2 extractable phosphorus concentration is driven by intense weathering and irreversible nutrient depletion^40^. Therefore, in this context phosphorus appeared to be a much better indicator of soil age (i.e., advancement of pedogenesis) than of fertility^36,40^. We thus suggest that phosphorus content can be used as a proxy for ecosystem development depending on long-term climatic stability (LS) rather than as an indicator of resource availability. Moreover, in our sampling area, the highest diversity indices were found on the all-slope reliefs up to 800 m a.s.l. These relic reliefs correspond to the oldest geomorphological surfaces of the Guiana Shield^41^ and are covered by a homogeneous soil system dominated by deep (geric) Ferralsols, characterised by chemical poverty and high clay content (>50%), which need a million years in a hot wet climate to develop^39,42^. Clearly, these small “mountain” forests benefited from very long-term soil and climatic stability and are considered as “refugia”, unlike the southern peneplains (i.e., multiconcave reliefs) that are believed to have been drier during the Pleistocene^35^. An alternative interpretation of the negative relationship between the concentration of phosphorus in the soil and alpha diversity could be that high-level disturbances that reduce diversity also cause more rapid biogeochemical cycling due to the increasing abundance of fast-growing early successional pioneers. However, the absence of a significant relationship between Urticaceae frequency and soil phosphorus concentration does not support this hypothesis.

We also observed that tree communities growing on steeply sloped reliefs hosted a high and regular frequency of early successional pioneer species (Fig. 2). This composition points to ecosystem functioning based on long-term stability but also on a regular moderate regime of local disturbances. Conversely, forests on plains and hilly reliefs appear to have undergone more irregular and spatially contrasted dynamics with periods of intense disturbances (marked by very high Urticaceae frequencies at a few sites) followed by long periods of slow dynamics (explaining the low rate of Urticaceae at most of these sites). The high but regular turnover of small “mountain” forests may be due to regular treefalls on slopes, to the size of the largest trees encountered on the mountains (see Supplementary Information Fig. S5) and, therefore, to the larger areas affected by individual treefalls, as observed in logged forests^43^. Higher exposure to dominant winds may explain the steady disturbance regime on higher reliefs compared to that on lower reliefs.

Finally, the strong correlation between tree diversity and understory diversity suggests that the latter guild also reaches maximum diversity when disturbance is intermediate, even if we failed to directly detect this maximum (because of the scarcity of data on the understory).

All these results agree with the hypothesis that the IDH effect is an important driver in old-growth Neotropical forests. These results are in agreement with the assumption of Connell^6^, particularly in ancient forests covering small mountains that have benefited from long-term climatic stability (as demonstrated by their soil properties) but which are also subject to a sustained regime of disturbance due to environmental conditions (slope, exposure, forest structure). This combination probably explains the high level of diversity reached by floristic communities in these landscapes. We can hypothesise that these mechanisms also may explain the diversity patterns in Atlantic forests in eastern Brazil, which share common features with Guianan forests in terms of climatic history and phylogenetic diversity^44^ and where maximum richness is also found in mid-altitude submountain forests^45,46^.

## Conclusion

We found a new indicator of disturbance regimes, relative frequency of Urticaceae trees, which is highly correlated with wood specific gravity (classical indicator of a disturbance regime) and canopy gaps fraction from LiDAR, but much easier to implement at large scale. We also found hump-shaped relationship between tree alpha diversity and Urticaceae frequency to confirm the validation of IDH at the regional scale in old and stable tropical forests. In fact, disturbance regimes explain a large proportion of variation in regional diversity in ancient forest ecosystems. Disturbances appear to have had a cumulative effect over time: intermediate disturbance regimes continuing over millennia enabled not only light-wood and light-demanding species to maintain themselves in the forest community, but also the development of a species-rich understory vegetation and a diversified assemblage of late-successional tree species. Our results agree with the assumption that high species diversity in the oldest Neotropical rainforests is the result of a combination of ancient diversification and long-term persistence of species, both leading to high species richness in regional pools, and recent ecosystem dynamics driving speciation^47^ to which contrasting forest landscapes under geomorphic control probably contribute^18,41^. Current climate change and human activities may be modifying recent ecosystem dynamics because of changes in the intensity and frequency of disturbances with an increase in the risk of drought^48^ and/or more frequent extreme precipitation events generating blowdowns^49^, but the consequences for forest diversity remain uncertain. Drought, timber harvest and fire events may open new successional pathways in the region and shift present forest functioning beyond a tipping point^50^. However, our empirical observations in the region suggest that a slight increase in disturbances caused by extreme precipitation events may trigger different responses depending on the geomorphological context, which reflects different levels of stability and different ecosystem dynamics. In this context, taking the current disturbance regime into account and including geomorphological landscape stratifications in climate-vegetation models may be an effective method for improving the prediction of local vegetation changes under climate change.

## Materials and Methods

### Study sites

French Guiana (4°13′N, 52°59′W) covers an area of approximately 85,000 km² in the eastern part of the Guiana Shield, and the altitude ranges between 0 m and 830 m above sea level. The climate is equatorial, with annual rainfall ranging from 4,000 mm in the northeastern portion to 2,000 mm in the southern and western portions, and the mean annual temperature is 26 °C. The length of dry season (i.e., number of consecutive months with less than 100 mm of precipitation) is two months in the north and three in the south. The main geological formations in French Guiana are 2.2–1.9 Ga plutonic and volcanic rocks.

**Figure 7:**
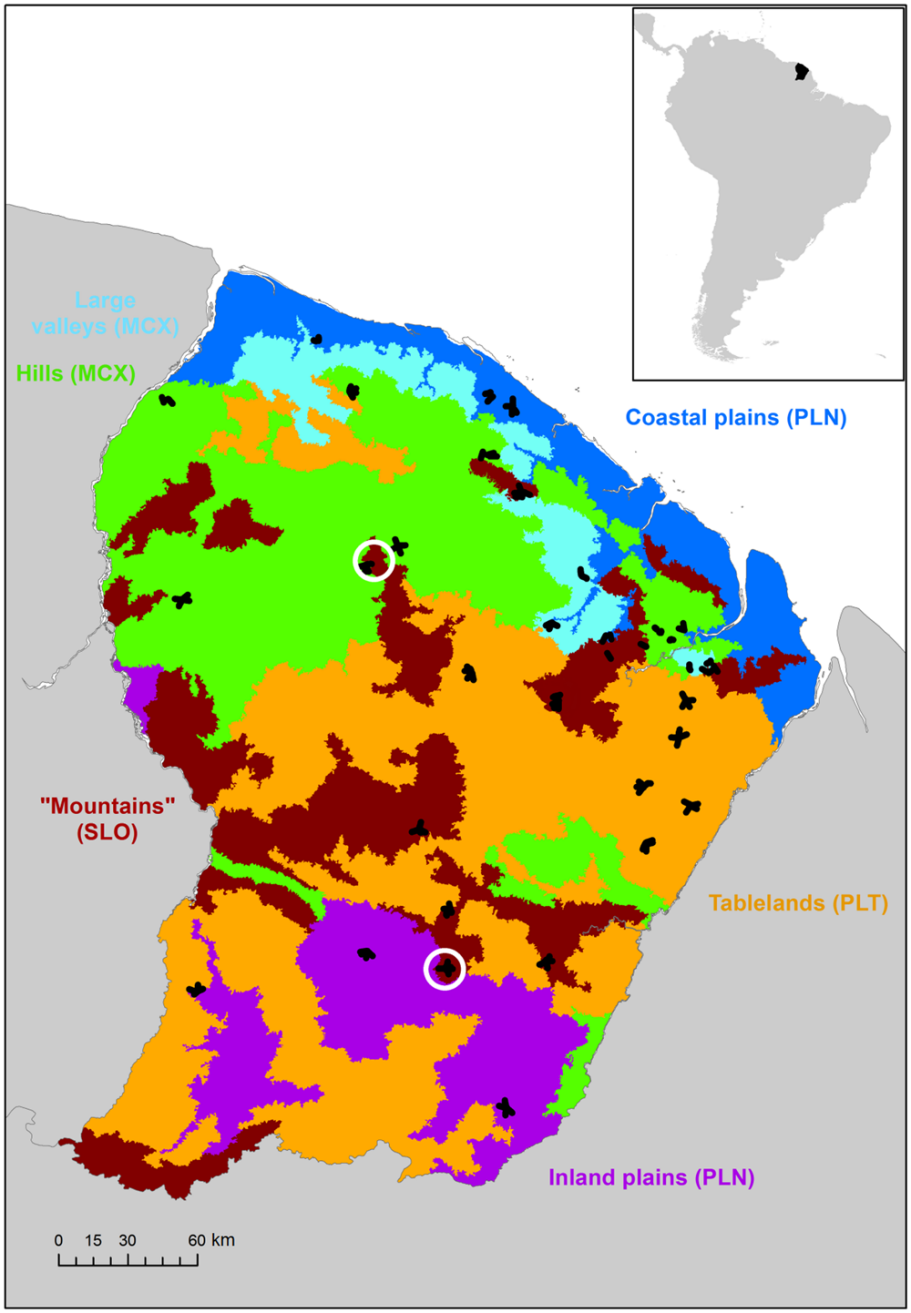
Location of the study sites across French Guiana. Transects are in black, focal sites with LiDAR cover are in white, and colours indicate the main geomorphological landscape categories across French Guiana. This map was modified from a previous version^53^ using ArcMap10.1 (http://esri.com). The abbreviations in brackets indicate the main landscape categories shown in the other figures.

Data on flora, fauna and the soil were collected at 33 sites in French Guiana (Fig. 7) between 2006 and 2015 by a multidisciplinary team of foresters, community ecologists, soil scientists, botanists and zoologists. Sites were selected in old-growth forests (excluding the man-modified coastal vegetation) across the entire territory that were representative of the different forest types^51^, climate variability^52^ within the region, and the diversity of geomorphological landscapes^53^, which influence floristic composition^18,39^. In this study, we distinguished four main categories of landscapes across French Guiana: (i) all-slope reliefs, also locally called mountains (SLO); (ii) plateau or tablelands (PLT); (iii) multiconvex reliefs shaped by successions of hills (MCX) associated with wide valleys (VLL); (iv) coastal plains (PLN) here grouped with multiconcave reliefs (MCV) corresponding to inland plains.

Data were collected along 3-km line transects chosen to capture local heterogeneity in terms of topography, geology, and geomorphology within the same type of landscape. Two to four transects per site were chosen as randomly as possible, depending on the local heterogeneity, field constraints and the extent of the landscape type. The line transects radiated from a central point (the campsite). The metadata are presented in Table SI1, and the sampling protocols are detailed hereafter.

### Tree community data

We inventoried all large trees with a diameter at breast height (DBH) >17.5 cm (excluding the young dominated strata and regeneration stand where competitive exclusion is still in progress), in a 20-m strip along the 3-km-long transects. Trees were identified using a vernacular nomenclature based on 232 local names corresponding to a species or group of species. Correspondences between vernacular nomenclature and taxonomy were established using independent double-blind determinations to control for botanical uncertainties^54^. We computed Fisher’s alpha diversity indices by simulating species composition while propagating the uncertainty of the vernacular nomenclature using Monte Carlo simulations^54^. The Fisher’s alpha diversity estimated using this method ranged from 134 to 194 with uncertainty ranging from 4% to 7% depending on the site.

We then computed the relative frequency of *Cecropia* and *Pourouma*, two genera belonging to the Urticaceae family. *Cecropia* comprise small-seeded, short-lived pioneers widely distributed throughout South America that are closely associated with secondary and disturbed forest; species of this genus are usually encountered in large natural treefall gaps^20^. They are well-known indicators of the earliest stage of forest succession after disturbances^21^. In French Guiana, the *Cecropia* genus comprises only eight species. Most of the ten species in the *Pourouma* genus^55^ are also small-seeded, short-lived pioneers that tend to replace *Cecropia* in small gaps. *Cecropia* and *Pourouma* species are very easily recognised by foresters and are rarely confused with species from other genera.

Following the methods of Molino and Sabatier^7^, we also computed the frequency of all light-demanding species having small seeds and light wood. The list of species of these authors was complemented by referring to a regional database containing functional traits^56^ and included 60 species from a total of 1,581 species inventoried in French Guiana^55^. We also computed the mean wood specific gravity using the global wood density database as reference^57^. These indicators were produced by simulating species composition from forest inventories using the same Monte Carlo process than for Fisher’s Alpha diversity^54^.

### Soil data

Soil samples were collected with a hand-auger at 450 locations along transects chosen to represent variability in elevation, geology and topographical positions. Core soil samples were collected at 20-cm intervals from the surface to a depth of 120 cm and dried as soon as possible. For each core, seven layers were described in the field (0–10 cm, 10–20 cm and then every 20 cm until 1.20 m) using a standardised protocol documenting soil moisture; colour; texture (by touch); quantity of roots; the nature and quantity of coarse fragments; and redox features. The field descriptions were used to classify the samples using principal component analysis (PCA), resulting in six main soil groups^39^ according to the World Reference Base nomenclature^58^: Ferralsols, Acrisols, Plinthosols, Cambisols, Arenosols and Podzols. We then computed the Euclidean distance between soil samples using the coordinates of the samples on the seven principal axes and computed the mean distance between samples within sites to provide an indicator of soil diversity. Sampling intensity varied between sites depending on field constraints, but we verified that the soil diversity indicator was not correlated with the number of samples (Table S1 - R² = 0). We also conducted chemical and physical analyses on composite samples representing the dominant soil type indicated by the PCA classification at each site. Composite samples contained two to five samples taken at the same site, at the same depth (0–20 cm) and of the same soil type. Sampling was completed by opening 11 soil pits (1 to 2 m deep), which provided supplementary material for laboratory analyses. Pit sampling and composite sampling provided direct measures of CEC, pH, sum of bases and phosphorus for 21 sites. All the soil samples were analysed at the CIRAD laboratory in Montpellier, France. Phosphorus was extracted with 0.03 M ammonium fluoride (NH_4_F) and 0.1 M hydrochloric acid (HCl) for 40 seconds. A 1:7 soil:solution ratio was used (Bray-2 method). CEC was determined using the ammonium acetate (pH 7) displacement method. The sum of bases means the sum of Ca, Mg, K, and Na, which were displaced and measured using inductively coupled plasma atomic emission spectrometry. Soil pH was measured at a 1:5 soil:deionised water ratio. Texture (i.e., relative proportions of sand, silt and clay contents) was determined using the pipette method.

### Understory vegetation inventories

At 12 of the 33 sites, understory vegetation was inventoried in 25 m² plots replicated in different topographic contexts along the line transects. In this study, we considered all understory plants, which included herbaceous forest plants, shrubs and vines that can flower within 3 m of the ground at the onset of sexual maturity and do not change the characteristic shapes of their leaves. Within this floral assemblage, we considered angiosperms and pteridophytes *sensu lato* (including lycophytes) with no height or diameter threshold. In each 5 × 5 m plot, all individuals were counted and identified to the species level. Individuals that could not be identified in the field were sampled and dried, and they were later identified at the French Guiana herbarium. In total, more than 800 vouchers were assembled, and most were deposited in the French Guiana herbarium collection. Depending on site heterogeneity and area, 14 to 44 plots were delimited at each site. A total of 305 plots hosted 9,361 individuals belonging to 494 different species, including lianas and epiphytes but excluding tree saplings. We computed Fisher’s alpha diversity for each site and checked that this indicator was not biased by the different sampling intensities (R² = 0 between the diversity indicator and the number of plots).

### LiDAR acquisitions

Eight supplementary 3-km-long transects were added at two focal sites (Trinité and Itoupé) in 2014 and 2015 along with LiDAR acquisition to measure disturbance intensity directly through canopy openness. At Trinité, two 3-km-long and 20-m-wide field transects were established on each side of a small mountain. The LiDAR acquisitions covered two areas that were 1-km-wide and 8-km-long. At Itoupé, LiDAR acquisition covered a single area 9-km-long and 8-km-wide. This area covered the four new transects on the two sides of the mountain (as at Trinité) and a central area corresponding to the summit of the mountain, where a network of transects had already been established in 2010.

LiDAR data were acquired by aeroplane at an altitude of ~600 metres above ground level. The on-board system comprised a scanning laser altimeter with a rotating mirror mechanism (Riegl LMS-Q560). The wavelength used was 1,550 nm. The scanning angle was +/−20°. The laser altimeter recorded up to 7 reflected pulses with a nominal precision of 10 cm. The final mean point density was 17 points/m² for a mean emitted density of 12 pulses per m^2^. Details on LiDAR data acquisition can be found on the geoportal of the laboratory (http://vmamapgn-test.mpl.ird.fr:8080/geonetwork/srv/eng/search). First returns were triangulated, and a 1-m raster was built by interpolation of the triangulated surface to obtain a canopy surface model. The digital terrain model was obtained in a similar way by interpolating the triangulated ground points. A canopy height model (CHM) was computed as the difference between the canopy surface model and the digital terrain model.

**Figure 8:**
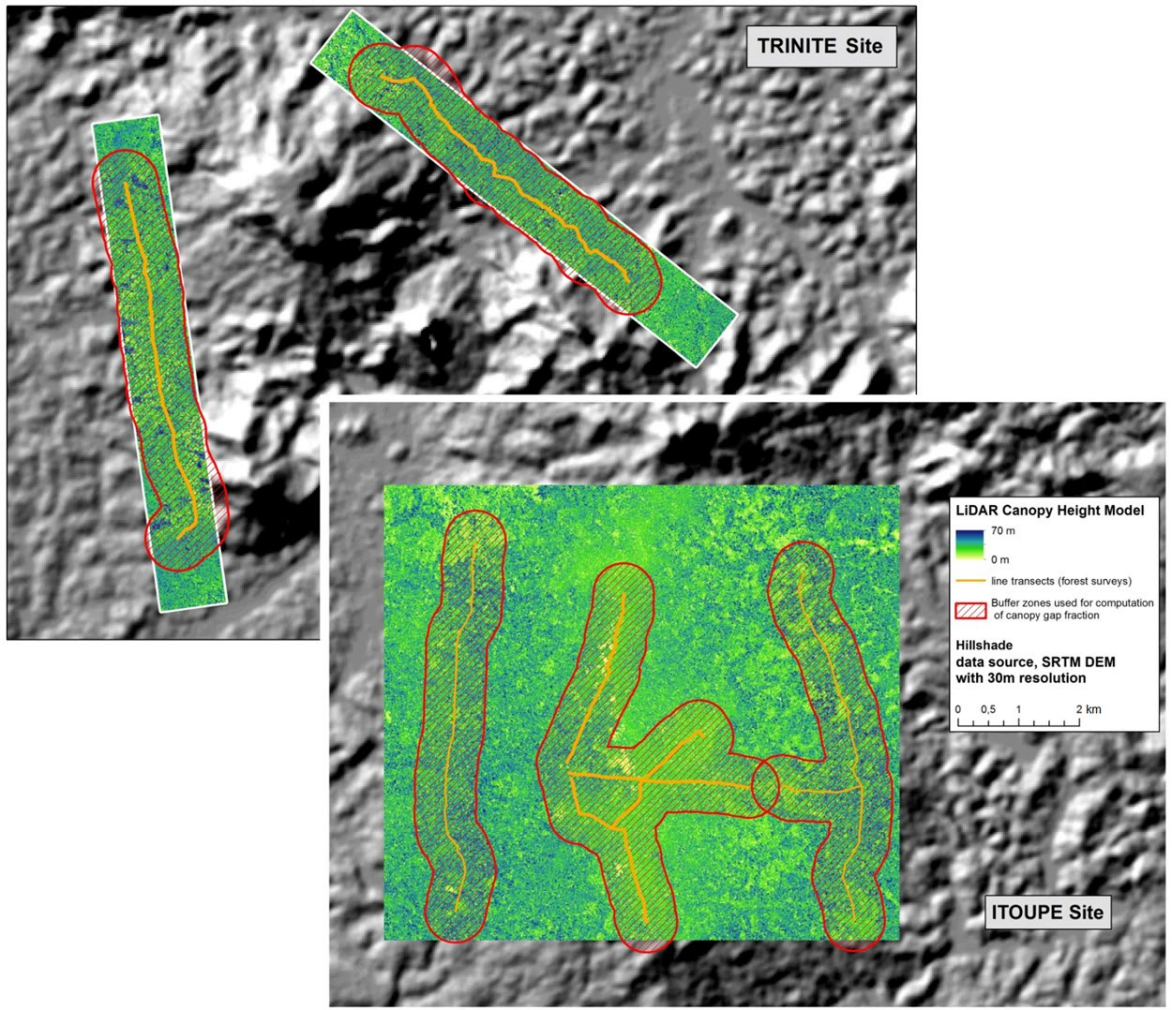
Canopy height model generated from LiDAR acquisitions at two focal sites. The percentages of gaps were computed for the five buffer zones (in red) surrounding the line transects (in orange) using ArcMap 10.1 (http://esri.com).

### Analyses

In a preliminary step, we checked the actual indicator value of Urticaceae frequency and the frequency of all pioneer species by testing the correlation with the surface area of gaps mapped at the two focal sites at which LiDAR data coupled with field transects were available (Supplementary Fig. S3). Gaps were defined from the canopy height model (CHM) with a 1-m cell size by applying different thresholds ranging from 2 m^59^ to 11 m^60^. We computed the fraction of canopy gaps in a 500-m wide buffer zone around each group of line transects (Fig. 8). These buffer areas were 7 km² in size on each mountainside and 10 km² in size on the summit. Using linear regressions, we then tested the correlation between gap fractions and Urticaceae frequency in the five sectors with LiDAR acquisitions.

We used a general linear model to explain spatial patterns of diversity using environmental variables via a stepwise process (both directions) and the Akaike information criterion (AIC) to select the best explanatory variables. We compared the effects of the selected variables using ANOVA (R stats MASS and ade4 packages). We also checked for spatial autocorrelations in predicted variables using variogram (semi-variance) analysis (R geoR package). In these models, we introduced quadratic factors for rainfall and geographic positions (i.e., squared terms) that were informative in previous studies^61,62^.

In the second step, we tested the relative influence of the potential drivers acting on regional species pool diversity for the following: (i) intermediate disturbance (ID) by introducing the relative frequency of Urticaceae and all pioneer indicators as well as their quadratic values; (ii) niche diversification (ND) by introducing soil diversity indices and the range of elevation as measures of spatial heterogeneity; and (iii) resource availability (RA) by introducing measures of soil texture and chemical analyses (clay content, CEC, sum of base, and Bray-2 extractable phosphorus). As we had a large number of potential predictor variables for a few observations, we first used Bayesian model sampling to select the predictor variables considering their posterior inclusion probabilities (package R bms). Then, we introduced the most frequently selected predictor variables in a GLM and stepwise algorithm to select the best model (i.e., that with the lowest AIC, using the glmulti package). Finally, to test the robustness of the model, especially the quadratic term of the model that caused the unimodal shape of the relationship, we built the following model using the selected predictor variables (equation 1):

$$AlphaDiv\approx N({\theta }_{0}+{\theta }_{1}\times Urti+{\theta }_{2}\times Urt{i}^{{\theta }_{3}}+{\theta }_{4}\times Rain+{\theta }_{5}\times Alt;{\sigma }^{2})$$

where θ_0_:θ_5_ and σ are the model parameters to be inferred with uninformative priors, *AlphaDiv* is the observed Fisher’s alpha diversity, *Urti* is the percentage of Urticaceae, *Rain* is the normalised annual precipitation and *Alt* is the normalised altitude. Because *AlphaDiv* is not measured but is estimated by simulating species composition from the vernacular nomenclature (see above), uncertainties of *AlphaDiv* were propagated through the model inference in a Monte Carlo scheme. The full model was then inferred using an adaptive form of Hamiltonian Monte Carlo sampling. Code was then developed using the R language and the Rstan package^63^.

We also tested the relationship between tree diversity indices and understory using Pearson’s product moment correlation coefficient, and we used the same modelling approach (i.e., selection of predictor variables, selection of the model and test of robustness) separately for understory vegetation diversity.

## Electronic supplementary material

Supplementary information
