# Author Correction: Disturbance Regimes Drive The Diversity of Regional Floristic Pools Across Guianan Rainforest Landscapes

**DOI:** 10.1038/s41598-018-24182-9

**Published:** 2018-04-12

**Authors:** Stéphane Guitet, Daniel Sabatier, Olivier Brunaux, Pierre Couteron, Thomas Denis, Vincent Freycon, Sophie Gonzalez, Bruno Hérault, Gaëlle Jaouen, Jean-François Molino, Raphaël Pélissier, Cécile Richard-Hansen, Grégoire Vincent

**Affiliations:** 1 AMAP, IRD, Cirad, CNRS, INRA, Université de Montpellier, Montpellier, France; 2 ONF Guyane, Département Recherche et Développement, Réserve de Montabo, 97307 Cayenne, French Guiana; 3 ONCFS, Direction de la Recherche et de l’Expertise, Campus agronomique, 97379 Kourou, French Guiana; 4 CIRAD, Forêts et Sociétés, Campus de Baillarguet, 34398 Montpellier, France; 5 IRD, Amap, Herbier de Cayenne, 97307 Cayenne, French Guiana; 6 CIRAD, EcoFoG, 97379 Kourou, French Guiana; 7 AgroParisTech, EcoFoG, 97379 Kourou, French Guiana

Correction to: *Scientific Reports* 10.1038/s41598-018-22209-9, published online 01 March 2018

In the original version of this Article, there were errors in Affiliation 1 which was incorrectly listed as ‘IRD, Amap, 34398, Montpellier, France.’ The correct affiliation is listed below:

AMAP, IRD, Cirad, CNRS, INRA, Université de Montpellier, Montpellier, France.

These errors have now been corrected in the PDF and HTML versions of the Article, and in the accompanying supplementary material.

