# Supplementary material for: Disturbance Regimes Drive The Diversity of Regional Floristic Pools Across Guianan Rainforest Landscapes
Source: Sci Rep. 2018 Mar 1;8:3872. doi: 10.1038/s41598-018-22209-9 (PMC5832822; doi:10.1038/s41598-018-22209-9)
Supplement: Supplementary file 1 — Supplementary information [file 41598_2018_22209_MOESM1_ESM.docx]

# DISTURBANCE REGIMES DRIVE THE DIVERSITY OF REGIONAL FLORISTIC POOLS ACROSS GUIANAN RAINFOREST LANDSCAPES

*Stéphane Guitet^1,2*^, Daniel Sabatier^1^, Olivier Brunaux^2^, Pierre Couteron^1^, Thomas Denis^3^, Vincent Freycon^4^, Sophie Gonzalez^5^, Bruno Hérault^6^, Gaëlle Jaouen^7^, Jean-François Molino^1^, Raphaël Pélissier^1^, Cécile Richard-Hansen^3^, Grégoire Vincent^1^*

^1^ AMAP, IRD, Cirad, CNRS, INRA, Université de Montpellier, Montpellier, France; ^2^ONF Guyane, Département Recherche et Développement, Réserve de Montabo, 97307 Cayenne, French Guiana ; ^3^ONCFS, Direction de la Recherche et de l’Expertise, Campus agronomique, 97379 Kourou, French Guiana ; ^4^CIRAD, UR Forêts et Sociétés, Campus de Baillarguet, 34398 Montpellier, France ; ^5^IRD, Herbier de Cayenne, 97307 Cayenne, French Guiana ; ^6^ CIRAD, UMR EcoFoG, 97379 Kourou, French Guiana, ^7^ AgroParisTech, EcoFoG, 97379 Kourou, French Guiana.

*corresponding author: [stephane.guitet@cirad.fr](mailto:stephane.guitet@cirad.fr)

## Supplementary information


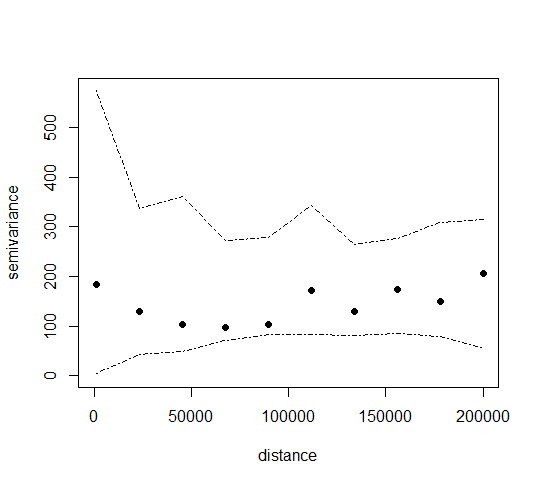


**Figure S1. Variogram of the alpha-diversity values on spatial locations with an envelope based on 999 permutations for significance**.


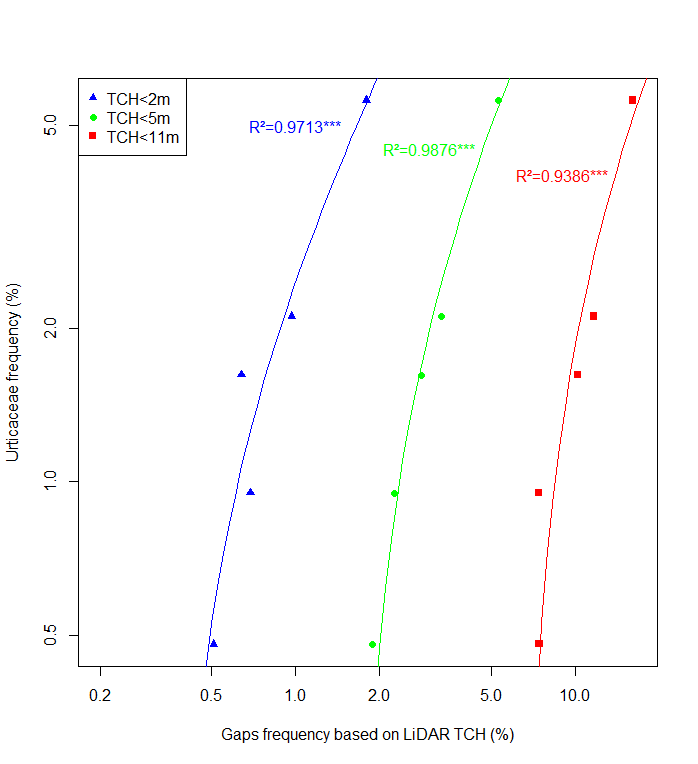

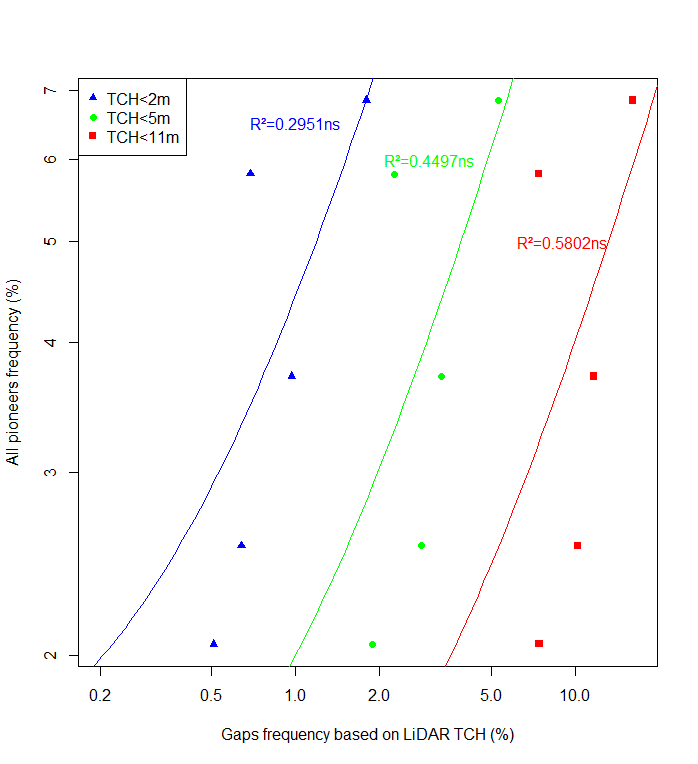


**Figure S2: Correlation between pioneer species (All species on the top and only Urticaceae on the bottom) frequency and percentage of gaps in the canopy measured from LIDAR with different threshold on the two focal sites.**


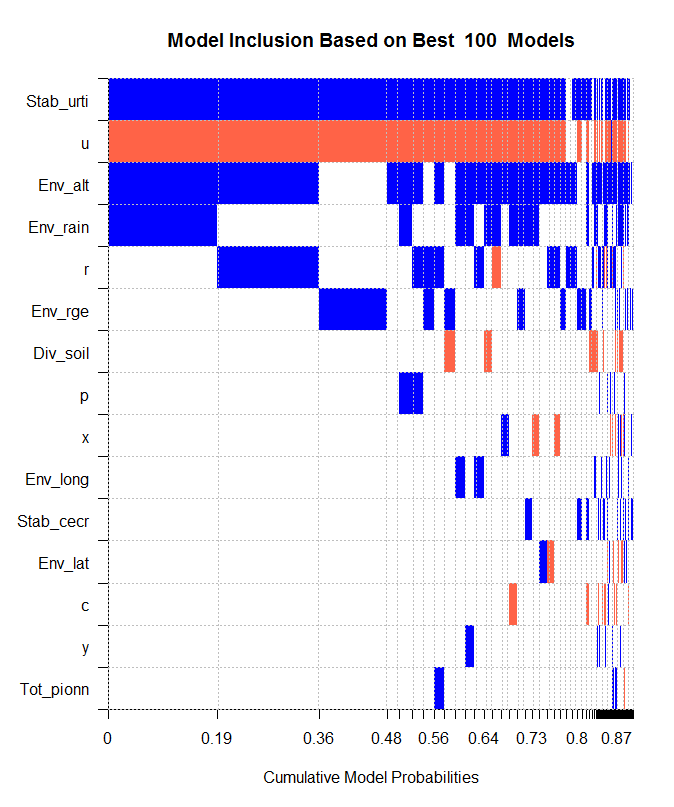

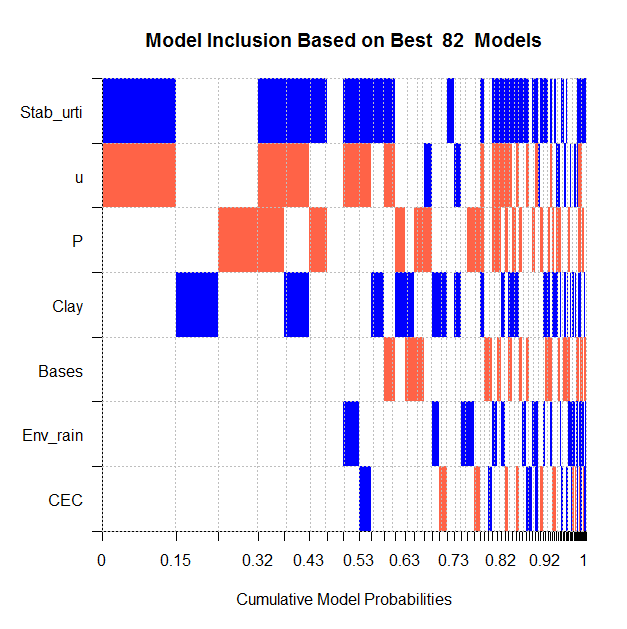


**Figures S3: Probability of inclusion of the environmental variables in the diversity prediction models, based on Bayesian approach.** Blue indicates a positive effect and red a negative effect. The variables are ranked according to performance. The width of the bars indicates the frequency of inclusion in the best models. The codes for the variables correspond to those in table SI2. u, r, p, c, x and y correspond respectively to the quadratic effect for Urticaceae frequency, rainfall, frequency of all pioneers, cecropia frequency, longitude and latitude. On the top, models with no soil variables, on the bottom, models including soil variables.


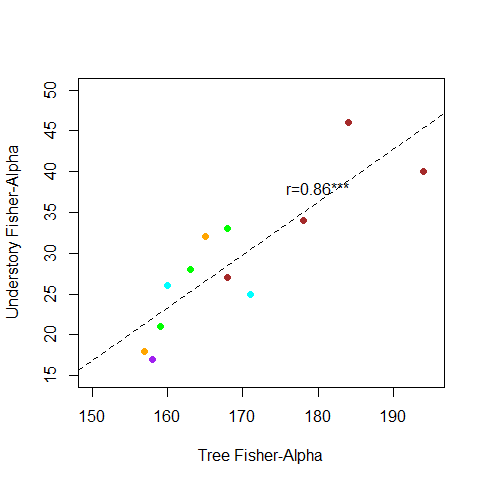


**Figure S4: Relationship between tree community alpha diversity and understory vegetation alpha-diversity at the site scale.** Colours indicate the relief categories (same codes as previous figures).


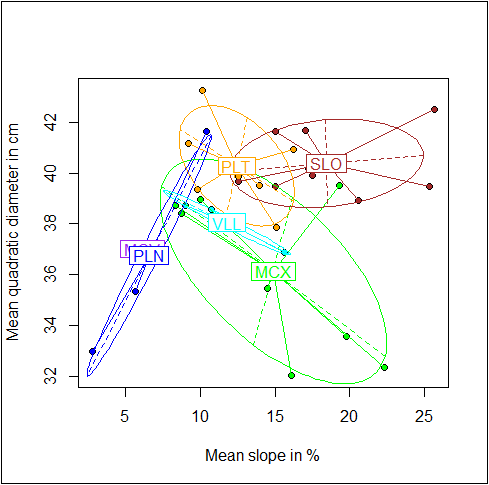


**Figure S5. Mean slope and mean quadratic diameter at the site scale in relation with the type of landscape:** mountains (SLO) combine the biggest trees with the steepest slopes which may explain larger treefall gaps and hence the regular intermediate disturbance regime.

**Table S1: Database** with diversity indices (for trees, soil, fauna, understory vegetation) and environmental variables (mean altitude, elevation range, rainfall, landscape type, mean slope, latitude and longitude) for each study site.

| ***Site*** | *Diversity indices* | | | | *Environmental and spatial variables* | | | | | | |
| --- | --- | --- | --- | --- | --- | --- | --- | --- | --- | --- | --- |
|  | *Div_*  *trees* | *Div_*  *soil* | *Div_*  *fauna* | *Div_*  *unders* | *Env_*  *alt* | *Env_*  *rge* | *Env_*  *rain* | *Env_*  *land* | *Env_*  *slop* | *Env_*  *lat* | *Env_*  *long* |
| **Aima** | 163 | 4.62 | 22.36 | 28 | 90 | 67 | 2695 | MCX | 8.39 | 246611 | 516825 |
| **Bago** | 171 | 3.31 |  | 25 | 110 | 70 | 3142 | VLL | 15.66 | 326441 | 504594 |
| **Bast** | 135 | 5.02 |  |  | 80 | 87 | 2477 | MCX | 16.07 | 146403 | 579995 |
| **Beim** | 168 | 3.2 | 22.59 | 33 | 110 | 49 | 2607 | MCX | 10.05 | 153223 | 493702 |
| **Cpar** | 144 | 5.86 |  |  | 45 | 35 | 2573 | PLN | 7.47 | 285914 | 581976 |
| **CriArm** | 177 | 2.17 | 25.66 |  | 80 | 79 | 2897 | PLT | 9.86 | 373236 | 404580 |
| **Croiss** | 156 | 1.85 | 22.58 |  | 130 | 90 | 2847 | PLT | 9.22 | 354390 | 387865 |
| **Csgk** | 159 | 5.53 | 23.33 |  | 45 | 41 | 2604 | PLN | 5.71 | 295921 | 577069 |
| **Eaub** | 167 | 5.03 |  |  | 70 | 80 | 2576 | MCX | 14.49 | 226520 | 584187 |
| **Gali** | 171 | 4.6 | 17.85 |  | 55 | 59 | 3099 | VLL | 10.73 | 312862 | 482893 |
| **Grill** | 147 | 4.21 |  |  | 30 | 46 | 3147 | PLN | 10.43 | 337150 | 477276 |
| **Hmata** | 164 | 3.34 | 24.02 |  | 105 | 75 | 2993 | PLT | 12.59 | 368012 | 434516 |
| **Iner** | 163 | 4.72 |  |  | 115 | 332 | 3156 | MCX | 19.31 | 369698 | 481674 |
| **Itou** | 194 | 3.81 | 19.73 | 40 | 670 | 556 | 2572 | SLO | 17.22 | 267617 | 334345 |
| **Kou** | 184 | 3.4 |  | 46 | 85 | 171 | 3143 | SLO | 17.01 | 381820 | 463286 |
| **Limo** | 170 | 5.2 | 23.66 |  | 225 | 81 | 2606 | SLO | 12.59 | 255676 | 394692 |
| **Man** | 171 | 2.29 | 21.99 |  | 50 | 77 | 3091 | PLT | 13.99 | 371085 | 450400 |
| **Nour** | 170 | 3.88 | 24.6 |  | 175 | 151 | 3030 | SLO | 15 | 315113 | 451721 |
| **Para** | 169 | 2.18 | 22.93 |  | 100 | 116 | 3030 | SLO | 15 | 314907 | 448097 |
| **PitArm** | 156 | 2.38 | 26.6 |  | 120 | 71 | 2636 | PLT | 10.16 | 352363 | 413243 |
| **PitBar** | 178 | 2.85 | 23.32 | 34 | 315 | 333 | 2876 | SLO | 25.67 | 268473 | 359560 |
| **Plomb** | 144 | 7.92 |  |  | 100 | 194 | 2800 | MCX | 19.8 | 285725 | 556402 |
| **Quar** | 159 | 6.14 |  | 21 | 145 | 188 | 3168 | MCX | 22.29 | 361858 | 478535 |
| **Regi** | 160 | 5.54 |  | 26 | 60 | 64 | 3149 | VLL | 9.01 | 376890 | 465524 |
| **RocK** | 157 | 3 | 23.67 | 18 | 205 | 89 | 2554 | PLT | 15.12 | 159359 | 324905 |
| **Sabl** | 134 | 4.16 |  |  | 30 | 19 | 2380 | PLN | 2.82 | 211555 | 606356 |
| **SautPar** | 165 | 4.04 | 18.93 | 32 | 130 | 132 | 2878 | PLT | 16.2 | 277668 | 462108 |
| **StMi** | 168 | 5.42 |  |  | 75 | 239 | 2955 | SLO | 17.5 | 299681 | 540028 |
| **Topo** | 168 | 4.09 | 23.57 | 27 | 255 | 284 | 2733 | SLO | 20.6 | 310821 | 337081 |
| **Tort** | 179 | 3.47 |  |  | 250 | 398 | 3149 | SLO | 25.33 | 345280 | 471738 |
| **Trini** | 168 | 5.35 | 23.5 |  | 155 | 149 | 2666 | MCX | 8.74 | 232445 | 507988 |
| **Waki** | 158 | 4.54 | 21.7 | 17 | 175 | 47 | 2517 | MCV | 5.19 | 232743 | 340931 |
| **Yaro** | 155 | 4.2 | 26.27 |  | 170 | 59 | 2574 | MCV | 7.12 | 293103 | 273779 |

**Table S1: Database (continued)** with disturbance indices (in order: frequency of Urticaceae pioneer species, only cecropia, all pioneer species, mean quadratic diameter, wood specific gravity) and environmental variables (mean altitude, elevation range, rainfall, landscape type, mean slope, latitude and longitude), resource availability (CEC in cmolc.kg-1, % clay, available phosphorus and sum of base in cmolc.kg-1) and sampling intensity for each site (for soil, trees and understory vegetation).

| ***Site*** | *Disturbance indices* | | | | | *Soil analyses* | | | | *Sampling intensity* | | |
| --- | --- | --- | --- | --- | --- | --- | --- | --- | --- | --- | --- | --- |
|  | *Stab_*  *urti* | *Stab_*  *cecr* | *Tot_*  *pionn* | *Str_*  *diam* | *Wsg* | *CEC* | *Clay* | *P* | *Bases* | *N_soil* | *N_tree* | *N_under* |
| **Aima** | 3.3 | 1 | 6.9 | 38.73 | 0.66 | 6.48 | 23 | 3.29 | 0.4 | 19 | 4568 | 21 |
| **Bago** | 5.2 | 1.7 | 5.4 | 36.86 | 0.63 |  |  |  |  | 11 | 1980 | 19 |
| **Bast** | 0.2 | 0.1 | 1.7 | 32.02 | 0.67 |  |  |  |  | 3 | 2608 |  |
| **Beim** | 1.6 | 1.6 | 3.6 | 38.98 | 0.66 | 7.62 | 32 | 1.54 | 0.43 | 18 | 5207 | 44 |
| **Cpar** | 0.4 | 0.1 | 7.2 | 37.07 | 0.67 | 8.74 | 23 | 1.27 | 0.66 | 6 | 2438 |  |
| **CriArm** | 1.9 | 1.4 | 4.2 | 39.38 | 0.65 | 10.51 | 66 | 1.12 | 0.63 | 16 | 5183 |  |
| **Croiss** | 4.8 | 0.2 | 10 | 41.16 | 0.63 | 8.1 | 47 | 1.05 | 0.41 | 12 | 3986 |  |
| **Csgk** | 3.7 | 0.9 | 11.5 | 35.35 | 0.64 |  |  |  |  | 21 | 5007 |  |
| **Eaub** | 1.7 | 0.7 | 4.8 | 35.47 | 0.66 | 7.19 | 26 | 1.68 | 0.83 | 18 | 4905 |  |
| **Gali** | 5.6 | 1.3 | 17 | 38.58 | 0.63 |  |  |  |  | 16 | 2861 |  |
| **Grill** | 1.2 | 0.7 | 2.9 | 41.64 | 0.66 |  |  |  |  | 8 | 1959 |  |
| **Hmata** | 1.1 | 0.5 | 3.3 | 39.88 | 0.67 | 9.16 | 55 | 1.12 | 0.89 | 9 | 5463 |  |
| **Iner** | 1.7 | 1.4 | 3.9 | 39.51 | 0.66 |  |  |  |  | 5 | 1869 |  |
| **Itou** | 4.5 | 2.7 | 8.6 | 40.20 | 0.62 | 14.69 | 53 | 0.63 | 1.45 | 28 | 5516 | 35 |
| **Kou** | 4.3 | 0.8 | 9.2 | 41.68 | 0.63 |  |  |  |  | 11 | 3419 | 25 |
| **Limo** | 4.3 | 0.4 | 7.3 | 39.69 | 0.61 |  |  |  |  | 16 | 4004 |  |
| **Man** | 2 | 0.6 | 2.7 | 39.53 | 0.65 | 8 | 48 | 1.12 | 0.45 | 14 | 3401 |  |
| **Nour** | 2.6 | 1.5 | 2.3 | 41.63 | 0.65 | 10 | 59 | 1.23 | 0.95 | 10 | 2465 |  |
| **Para** | 4.1 | 0 | 9.9 | 39.47 | 0.65 |  |  |  |  | 7 | 1985 |  |
| **PitArm** | 2.4 | 0.7 | 7.1 | 43.28 | 0.64 | 10.45 | 60 | 0.98 | 0.62 | 12 | 4054 |  |
| **PitBar** | 2.3 | 0.2 | 3.7 | 42.50 | 0.64 |  |  |  |  | 16 | 4069 | 16 |
| **Plomb** | 0.1 | 0.1 | 3.4 | 33.56 | 0.68 | 9.55 | 53 | 2 | 0.75 | 10 | 3998 |  |
| **Quar** | 0.2 | 0.1 | 2.4 | 32.34 | 0.69 | 4.6 | 8 | 2.25 | 0.65 | 15 | 2638 | 17 |
| **Regi** | 3.7 | 3.2 | 4.1 | 38.73 | 0.66 | 6.4 | 21 | 1.3 | 0.7 | 15 | 1958 | 14 |
| **RocK** | 2.1 | 0.6 | 3.5 | 37.88 | 0.64 |  |  |  |  | 16 | 4411 | 34 |
| **Sabl** | 0 | 0 | 2.8 | 32.97 | 0.67 | 16.14 | 5 | 3.28 | 3.24 | 1 | 1627 |  |
| **SautPar** | 3.9 | 1.1 | 5.8 | 40.95 | 0.64 | 10.54 | 50 | 0.98 | 0.68 | 20 | 4423 | 21 |
| **StMi** | 3.5 | 3.2 | 3.6 | 39.92 | 0.64 |  |  |  |  | 11 | 3731 |  |
| **Topo** | 2.7 | 0.4 | 8 | 38.93 | 0.63 | 12.09 | 59 | 1.12 | 1.89 | 23 | 4603 | 20 |
| **Tort** | 1.8 | 1.8 | 1.8 | 39.48 | 0.65 | 16.44 | 73 | 0.75 | 0.65 | 7 | 1900 |  |
| **Trini** | 1 | 0.3 | 4.3 | 38.42 | 0.66 | 3.29 | 16 | 2.03 | 0.64 | 17 | 5365 |  |
| **Waki** | 1.4 | 0.5 | 4.1 | 36.84 | 0.64 | 3.31 | 15 | 4 | 0.46 | 25 | 5106 | 39 |
| **Yaro** | 6.5 | 1.5 | 15.8 | 37.18 | 0.60 | 4.51 | 26 | 3.4 | 0.62 | 22 | 3965 |  |
